# Macro-Process of Past Plant Subsistence from the Upper Paleolithic to Middle Neolithic in China: A Quantitative Analysis of Multi-Archaeobotanical Data

**DOI:** 10.1371/journal.pone.0148136

**Published:** 2016-02-03

**Authors:** Can Wang, Houyuan Lu, Jianping Zhang, Keyang He, Xiujia Huan

**Affiliations:** 1 Key Laboratory of Cenozoic Geology and Environment, Institute of Geology and Geophysics, Chinese Academy of Sciences, Beijing 100029, China; 2 University of Chinese Academy of Sciences, Beijing 100049, China; 3 Center for Excellence in Tibetan Plateau Earth Science, Chinese Academy of Sciences, Beijing 100101, China; University of Oxford, UNITED KINGDOM

## Abstract

Detailed studies of the long-term development of plant use strategies indicate that plant subsistence patterns have noticeably changed since the Upper Paleolithic, when humans underwent a transitional process from foraging to agriculture. This transition was best recorded in west Asia; however, information about how plant subsistence changed during this transition remains limited in China. This lack of information is mainly due to a limited availability of sufficiently large, quantified archaeobotanical datasets and a paucity of related synthetic analyses. Here, we present a compilation of extensive archaeobotanical data derived from interdisciplinary approaches, and use quantitative analysis methods to reconstruct past plant use from the Upper Paleolithic to Middle Neolithic in China. Our results show that intentional exploitation for certain targeted plants, particularly grass seeds, may be traced back to about 30,000 years ago during the Upper Paleolithic. Subsequently, the gathering of wild plants dominated the subsistence system; however, this practice gradually diminished in dominance until about 6~5 ka cal BP during the Middle Neolithic. At this point, farming based on the domestication of cereals became the major subsistence practice. Interestingly, differences in plant use strategies were detected between north and south China, with respect to (1) the proportion of certain plant taxa in assemblages, (2) the domestication rate of cereals, and (3) the type of plant subsistence practiced after the establishment of full farming. In conclusion, the transition from foraging to rice and millet agriculture in China was a slow and long-term process spanning 10s of 1000s of years, which may be analogous to the developmental paths of wheat and barley farming in west Asia.

## Introduction

Plants are a critical component of human existence, both today and in the past. In the last 10 years, studies have documented that the long-term history of human-plant interaction dates back to the Pliocene [1–5]. This discovery indicates that humans have been exploiting plants for millions of years. Although plants may be used for a variety of purposes, such as fuels, tools, and building materials, their use as food represents a critical resource for human subsistence.

The use of plants as foods developed gradually, but dramatically changed in the Pliocene in parallel to advances in human evolution [5]. In general, the pattern of simple and random plant gathering was part of the lifestyle of early hominids until the Middle Paleolithic. During this period, the directional collection of certain plant foods was performed [6–9]. However, the use of plant foods became more sophisticated during the Upper Paleolithic, being characterized by intensive foraging, processing, and storing of a wide range of edible wild plants, especially grass seeds [8–14]. Yet, it is possible that this broad spectrum plant collecting emerged much earlier, around 250,000 years ago [15]. Following a long period during which people trialed wild plant cultivation, a new stage of plant use arose at the beginning of Holocene. This period is referred to as “low-level food production” and preceded the establishment of intensive agriculture. Low-level food production involved the practice of farming based on the domestication of cereals with the continued use of various wild plant foods [16].

Since the Upper Paleolithic, past plant subsistence appeared to noticeably transition from foraging to a cereal-based farming economy, i.e., the origin of agricultural process. This process has been best described for western Asia, where intensive archaeological/archaeobotanical studies have been performed for several decades (e.g. [17–25]). However, there is a lack of reliable archaeobotanical data from the indigenous origin center of agriculture in China. This lack is due to the long time paucity of scientific collecting techniques and analytical methods for ancient floral remains in China [26–28]. Fortunately, since the 21st century, archaeobotanical research has rapidly advanced in Chinese archaeology. Consequently, increasing numbers of studies have applied various systematic archaeobotanical approaches. Such approaches include the flotation technique, starch grain and phytolith analysis. As a result, valuable data have been obtained, particularly with respect to the early stages of the transition to agriculture (e.g. [29–41]). Thus, this information could contribute towards improving our understanding about the history of plant-based subsistence strategies during the shift towards agricultural processes in China.

In the last 8 years, a number of studies based on integrated archaeobotanical evidence have broadly delineated the changing patterns of plant food use during the origin of agricultural practices in China [26, 30, 31, 42–46]. However, these studies lacked quantitative data, and could not be validated. Therefore, in this study, we first systematically compiled an available archaeobotanical database of China generated from interdisciplinary approaches, dating between ca. 30,000 and 5,000 years ago. Subsequently, we quantitatively analyzed these datasets, along with other evidence, to reconstruct how plant subsistence changed from the Upper Paleolithic to the Middle Neolithic periods. Our results are expected to improve our understanding about how plant use changed from foraging to agriculture in China.

## Materials and Methods

The archaeobotanical data used here were obtained by a review of the relevant literature, including published research papers, reports, and completed dissertations. Data dated after ca. 5 ka cal BP were not included in the current study, as agriculture was well-established at this point. The data used in this study were mainly obtained from the analytical results of plant macro-remains and starch granules, supplemented by some phytolith data. Among the multiple forms of data in the original references, only the values of absolute amounts for certain plant remains were included in the database, and used as basis for our quantitative analysis (see S1 Dataset).

The collected data were from provenances that had clear age ranges determined by radiocarbon dating and features of cultural remains. These data were derived from systematic studies by well-trained professionals, ensuring that identified plant remains were of high quality. All data labeled as doubtful or unidentifiable in the sources were excluded from our analysis. The macro-botanical remains from Kuahuqiao and Bashidang sites [47, 48] were collected unsystematically during excavations, with hand picking by the naked eye and the occasional use of flotation methods; thus, these datasets should have been excluded from our analysis. However, due to the large quantity and diversity of species identified at these 2 sites, these data included as assisting materials in the discussion (but were not directly used in our analysis). In addition, despite some studies applying standard archaeobotanical methods, only some of the original data were published for certain plants (e.g. [49–51]), resulting in bias being generated among plants during statistical processing. Consequently, these data were excluded in the analysis.

**Fig 1:**
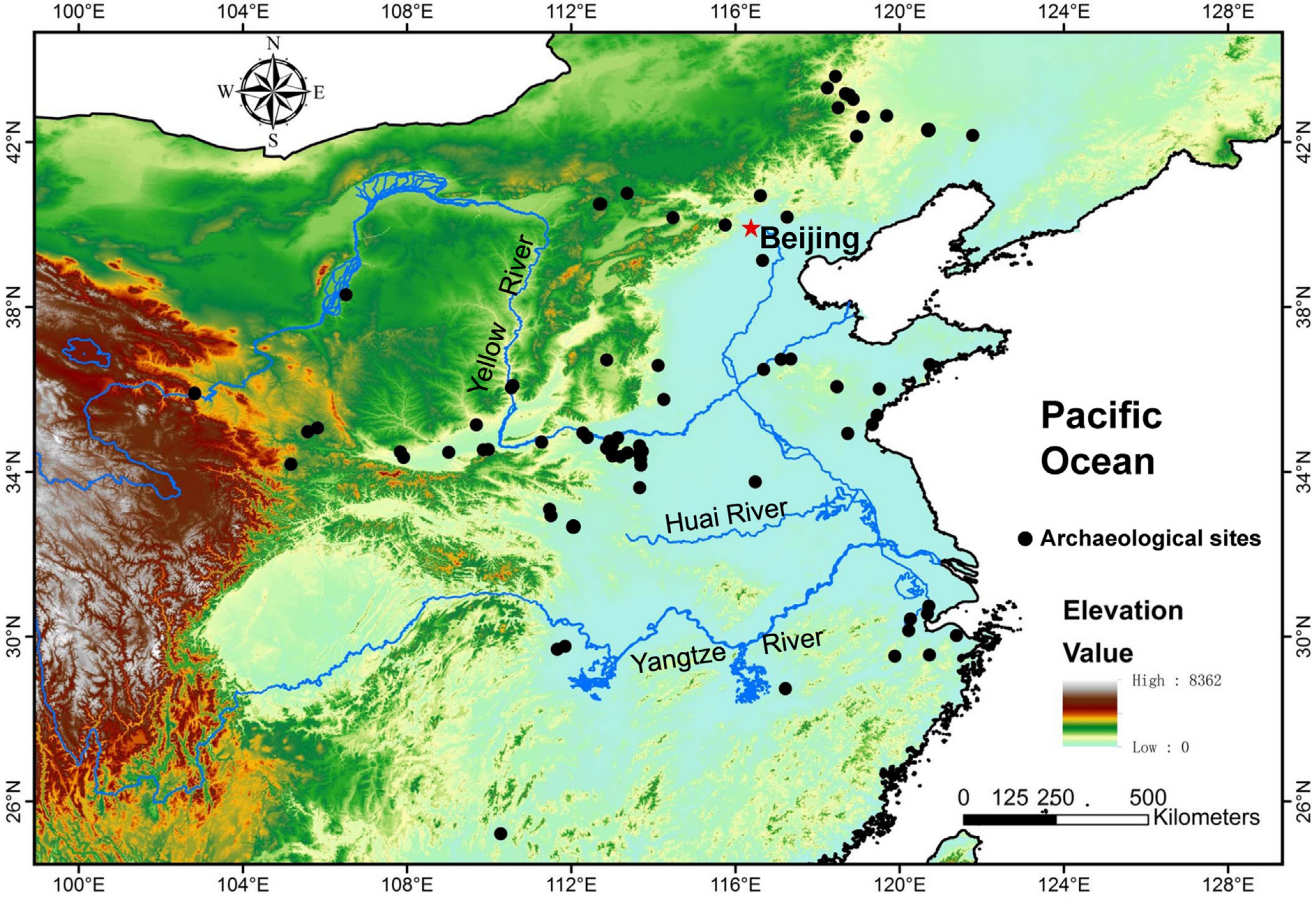
Locations of the archaeological sites in China from which archaeobotanical data were used in the current analysis. (map modified from Grass GIS; https://grass.osgeo.org/).

**Fig 2:**
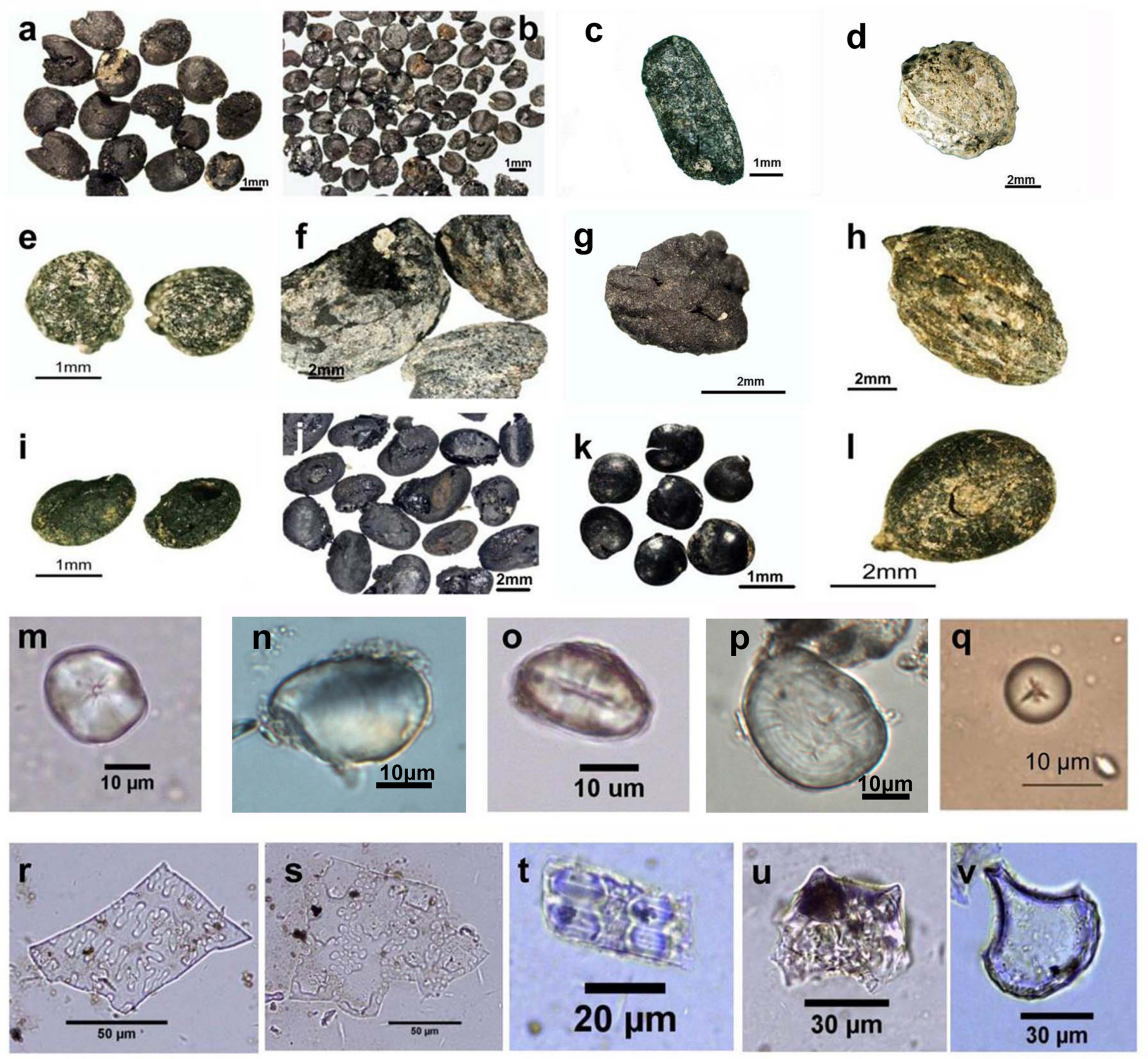
**Common plant remains excavated from Chinese archaeological sites, including plant macro-remains of (a) *Panicum miliaceum* L.; (b) *Setaria italica* Beauv.; (c) *Oryza sativa* L.; (d) *Celtis koraiensis* Nakai.; (e) *Broussonetia papyrifera* L. (f) *Quercus* sp.; (g) *Amygdalus persica* L.; (h) *Ziziphus jujuba* Mill. var. spinosa Hu; (i) *Melilotus* sp.; (j) Fabaceae; (k) *Chenopodium* sp.; (l) *Vitis* sp., starch granules (m) millets; (n) roots and tubers; (o) Triticeae; (p) food legumes; (q) acorns and phytoliths (r) η type from husks of common millet; (s) Ω type from husks of foxtail millet; (t) parallel-bilobe from rice leaf/stem; (u) double-peaked from rice husk; (v) and bulliform from rice leaves.** These photos of plant remains have not been previously published.

This study used archaeobotanical data derived from 88 archaeological sites, which were primarily located in northern China (Fig 1; S1 Dataset). A total of 90 plant families, 151 genera, and 127 species were detected at these sites, revealing a high taxonomic diversity of plants used through time (Fig 2).

The prehistoric culture of China may be broadly divided into 2 systems since the Upper Paleolithic; namely the North and South China systems. These 2 systems are characterized by different styles of stone tools, pottery, plant domestication, and agriculture [30, 52–54]. Therefore, we investigated plant use strategies of the 2 regions separately in the current analysis. Combining the academic opinions of previous studies [26, 31, 44] and the data used in the current study, the stages of development in plant use were broadly determined as: (1) ca. 33,000~19,000 cal yr BP, Upper Paleolithic; (2) ca. 14,000~9,000 cal yr BP, transitional phase from Paleolithic to Neolithic; (3) ca. 9,000~6,000 cal yr BP, Early Neolithic, within which the period between 7,000 and 6,000 cal yr BP was also considered for South China; and (4) 6,000~5,000 cal yr BP, Middle Neolithic. Although the data available for certain regions and periods are limited, we believe sufficient information was available to trace the macro-process of past plant subsistence.

The relative percentage and ubiquity may be used to analyze these data for each region and period. The relative percentage was used to detect how the proportions of exploited plants changed over time in past subsistence practices. Only data on plant macro-remains and starch granules were subjected to relative percentage analysis. The relative percentage analysis was calculated by n_1_/N_1_×100%, where n_1_ is the number of each plant group/type and N_1_ is the total number of all plant groups/types. Ubiquity, which is also called “presence analysis,” is frequently used to investigate the importance of certain plants in subsistence practices [26, 55, 56]. This analysis was conducted here with slight modification, in which sites represented the basic analytical units, rather than samples. Ubiquity was calculated by n_2_/N_2_×100%, where n_2_ is the number of sites in which certain plants were found and N_2_ is the total number of sites during certain periods. There were insufficient phytolith data for the relative percentage analysis. Furthermore, phytolith data were only used in the ubiquity analysis when the same sites also had macro-botanical and/or starch data. Thus, the archaeobotanical data from 84 sites of the 88 sites were ultimately used in the quantitative analysis. Ten of these sites were situated in South China and of these 74 sites were situated in North China.

Here, we mainly focus on the broad trajectories to the onset of full agriculture, which may be expressed as the transition from the gathering of wild plants to the cultivation of cereals as the major subsistence. Therefore, we do not focus on the use of every specific plant species. Thus, in the analysis, the plant species were separated into 6 groups: underground storage organs (USOs), fleshy fruits & nuts, beans, grasses, weeds, and cereals. Grasses included cereals and other Poaceae plants. Cereals (including wild and domesticated millets, rice, and Job’s-tears) were considered the basis of farming crops. Weeds may have been intentionally collected during the early stages of farming; however, they were commonly considered as arable accompanying species with cereals in farmland in the archaeobotanical records. Thus, weeds may be used as an indicator for agricultural production status. Other plant taxa were generally regarded as edible wild resources exploited by humans in the past. The relative percentage and ubiquity were then applied to determine the proportion or significance of certain types of plants used during past plant subsistence strategies. For example, in North China, archaeobotanical data dating in 9~6 ka cal BP derived from 40 sites. Within these data, there were a total of 18,791 plant macro-remains in which 3,856 cereals (rice, millets, etc.) were identified, then the proportion of cereals in macrobotanical remains was 21% (3,856/18,791×100%) during 9~6 ka cal BP. There were 15 sites in which macro-remains or starch grains of common millet were found; Cishan, Tanghu, and Xihe sites had macro-botanical and/or starch data without common millet, which was found in phytolith data of each site. Thus, common millet was present in 18 sites, and its ubiquity was 45% (18/40×100%).

## Results

### Past plant use in South China

The earliest evidence of plant use in South China derived from the Xianrendong and Diaotonghuan sites. At these sites, starch grains recovered from 2 shell tools were mostly identified as the tribes Paniceae and Triticeae, dating to 20~19 ka cal BP [57]. Thus, grasses use activity in South China extends back to the Last Glacial Maximum (LGM, 26.5~19 ka cal BP).

**Fig 3:**
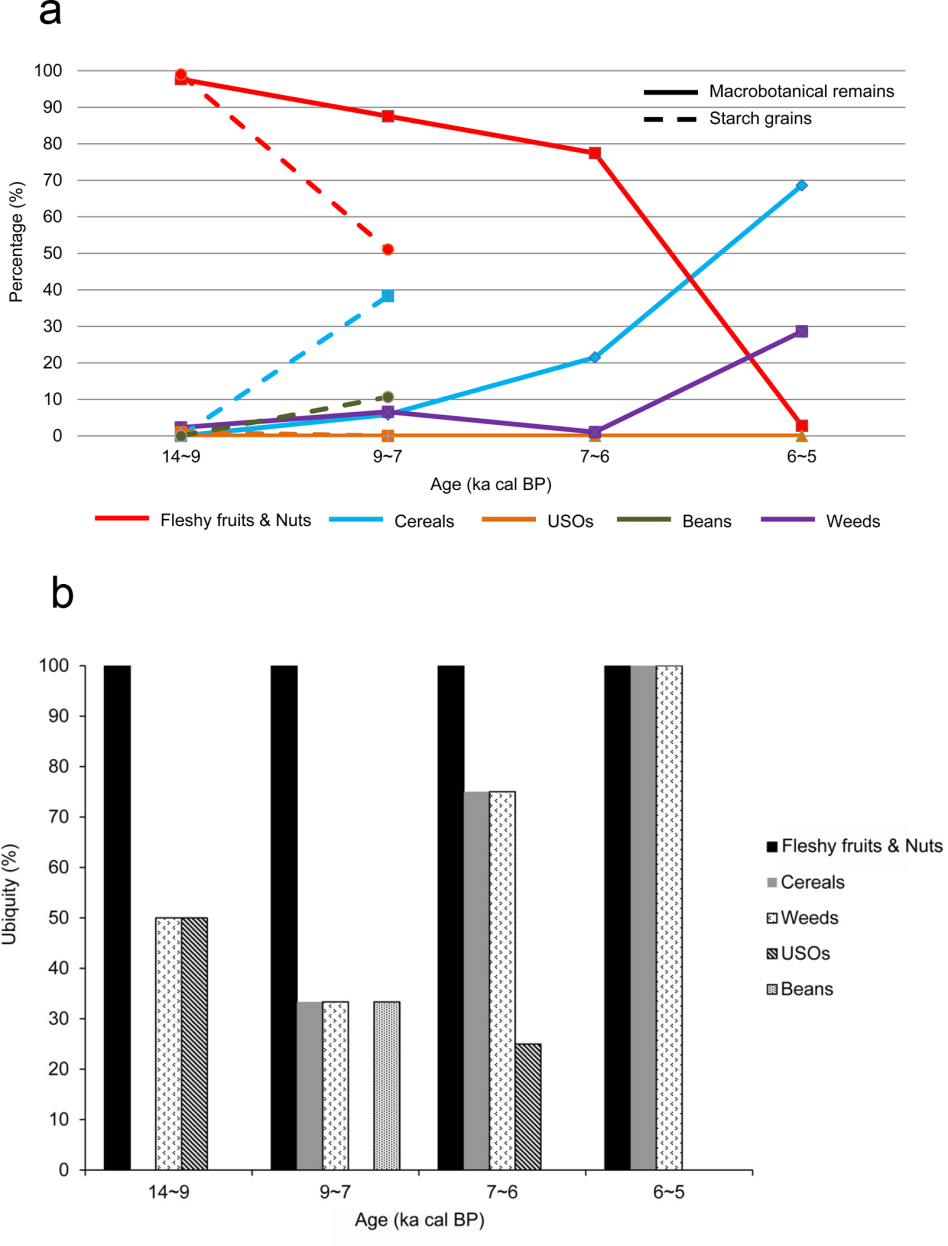
Relative percentage (a) and ubiquity (b) of the 5 plant groups in South China between 14 and 5 ka cal BP.

The relative percentage and ubiquity of the 5 plant groups in South China from ca. 14 to 5 ka cal BP were partitioned into 4 stages of agricultural transition (Fig 3). However, archaeobotanical information was absent from 19 to 14 ka cal BP in South China. Fleshy fruits and nuts (e.g., *Celtis sinensis*, *Prunus davidiana*, Fagaceae, *Trapa* sp.) consistently accounted for the greatest percentage of plants in the transitional phases and Early Neolithic period (78%–98% in macrobotanical remains and 51%–99% in starch grains). However, the percentage representation of this group sharply decreased during the Middle Neolithic, dating to 6~5 ka cal BP (3% in macrobotanical remains). Cereals found in South China included rice (*Oryza* sp.), foxtail millet (*S*. *italica*), and Job’s-tears (*Coix chinensis*), in which rice was the primary cereal detected, while the other two cereals were sporadically detected. The percentage representation of these all 3 cereals gradually increased through time, and became dominant in the Middle Neolithic (69% in macrobotanical remains). A similar trend was also detected for weeds, which mainly consisted of sedges (*Cyperus* sp., *Scirpus* sp., *Juncellus* sp.), and annual and perennial herbs (*Setaria* sp., *Digitaria* sp., *Polygonum* sp., *Humulus* sp., *Potamogeton* sp.). However, the percentage of USOs (e.g., *Dioscorea* sp.) and beans (e.g., *Vigna angularis*) was considerably lower than the other groups, and may not have been regularly used.

The percentage ubiquity of fleshy fruits and nuts was 100% for all four transition stages (Fig 3B). The ubiquity of cereals increased from the Early Neolithic (33%) to the Middle Neolithic (100%), with the same pattern being detected for weeds. USOs were occasionally used in 14~9 and 7~6 ka cal BP, with 50% and 25% ubiquity, respectively. Beans only appeared during 9~7 ka cal BP, with 33% ubiquity.

**Fig 4:**
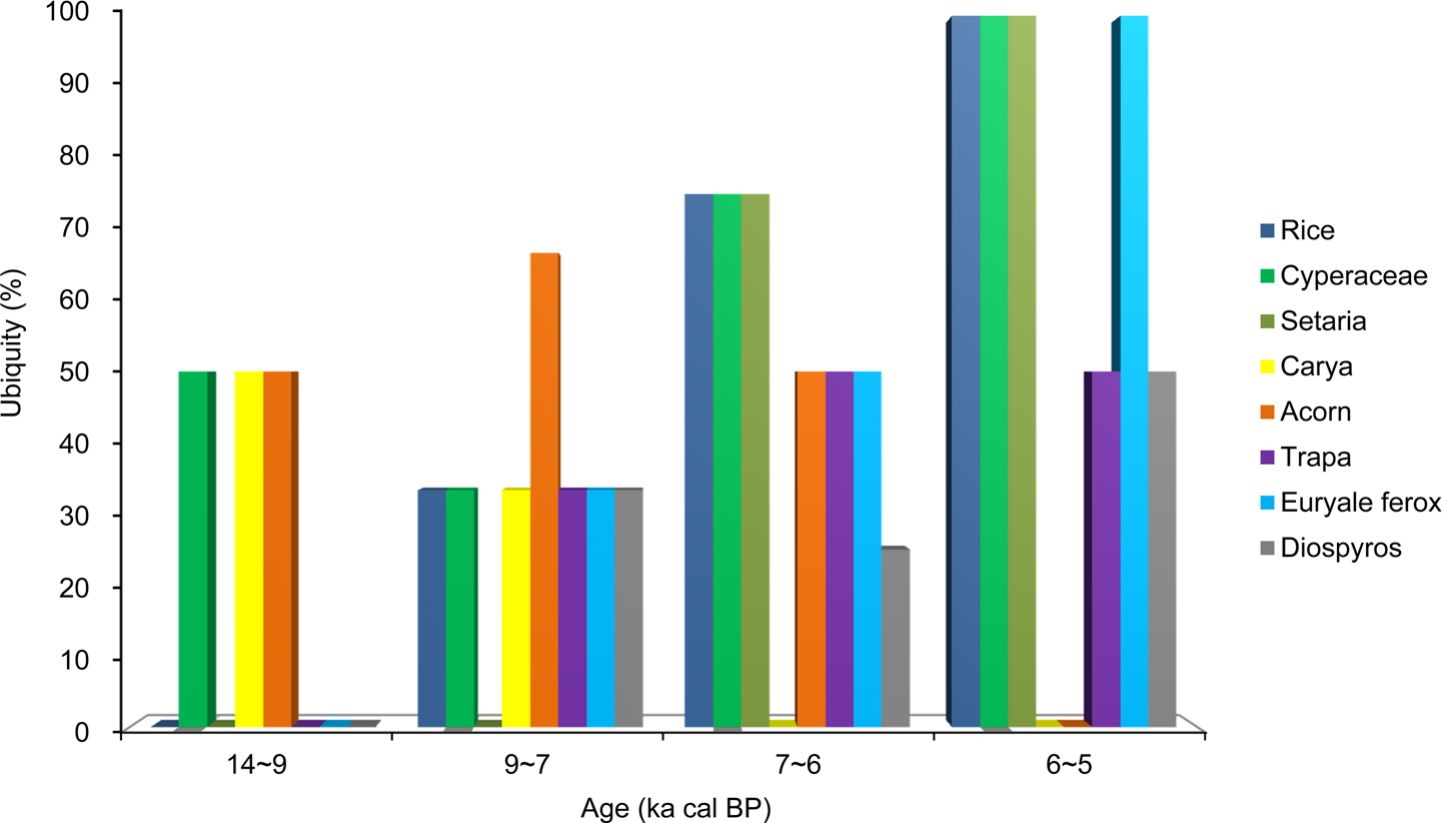
Ubiquity of some important plant species used in South China between 14 and 5 ka cal BP.

In South China, the ubiquity of acorns indicated that they were used as staples in the early stages of transition, particularly between 9 and 7 ka cal BP (Fig 4). From 7 to 5 ka cal BP, the ubiquity of rice, Cyperceae, and *Setaria* increased, whereas that of acorns disappeared (Fig 4). In contrast, the ubiquity of other edible wild plants (such as *Trapa*, *E*. *ferox*, and *Diospyros*) remained constant, representing important food resources.

**Fig 5:**
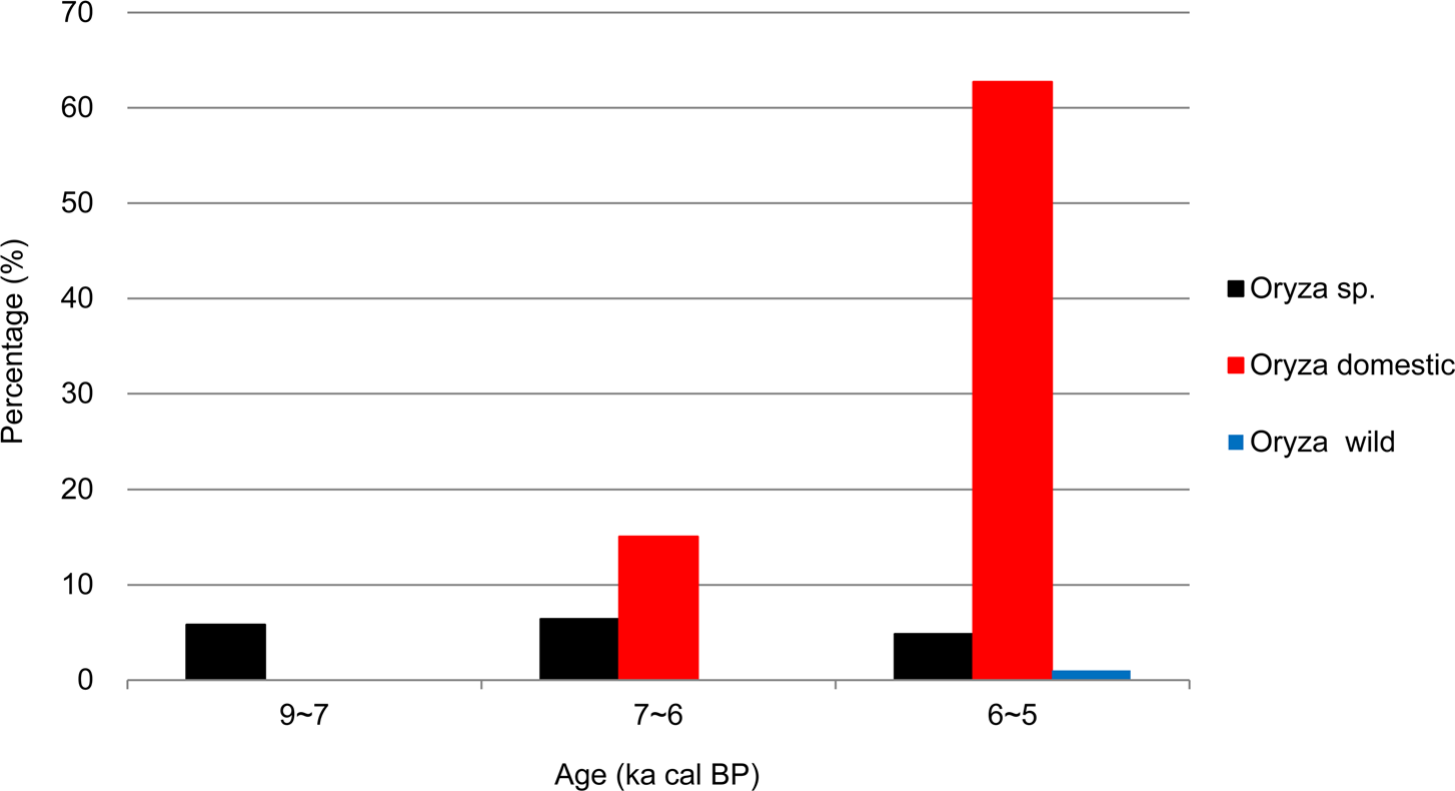
Relative percentage of genus *Oryza* plants between 9 and 5 ka cal BP.

The macrobotanical data analysis reflected the results obtained for rice domestication in South China (Fig 5). During 9~7 ka cal BP, the remains of rice were detected without any signs of domestication. From 7 to 6 ka cal BP, domesticated rice was identified, but only represented 15% of all analyzed rice. Between 6 and 5 ka cal BP, the amount of domesticated rice significantly increased to 63%.

### Past plant use in North China

**Fig 6:**
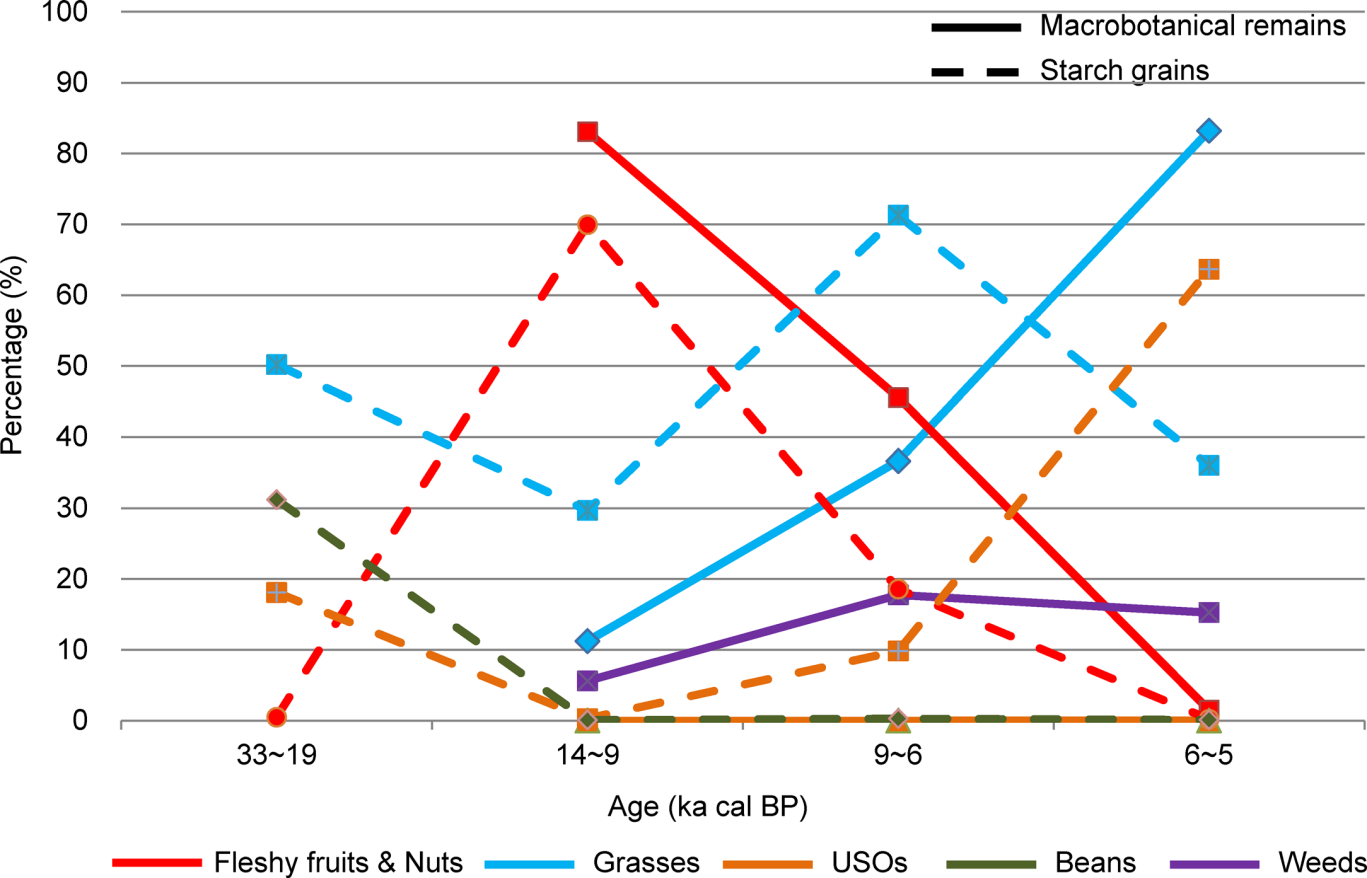
Relative percentage of the 5 plant groups in North China between 33 and 5 ka cal BP.

The percentage of the 5 plant groups in North China between ca. 33 and 5 ka cal BP were divided into 4 time intervals (Fig 6). However, archaeobotanical information was absent from 19 to 14 ka cal BP in North China. Information about plant foraging during the Upper Paleolithic (33~19 ka cal BP) was obtained from the starch grain analysis conducted on stone artifacts. Recovered starch grains were dominated by grasses taxa (50%), primarily the tribes Triticeae (40%) and Paniceae (9%), followed by beans of the Leguminosae family (31%), USOs (18%; including yams (*Dioscorea opposita*) (12%), cattail rhizomes (*Typha* sp.) (3%), snakegourd root (*Trichosanthes kirilowii*) (2%), and lily (*Fritillaria/Lilium* sp.) (1%)), and nuts (*Castanea/Quercus* sp.) (1%).

During the transitional period (14~9 ka cal BP), fleshy fruits and nuts dominated the assemblages (83% in the macrobotanical remains and 70% in starch grains), followed by grasses (11% in the macrobotanical remains; 29.7% in starch grains, 19.4% of which were millet), weeds (6% in the macrobotanical remains), and USOs and beans (0.3% and 0.1% in starch grains, respectively). Between 9 and 6 ka cal BP, the amount of fleshy fruits and nuts declined; however, they still accounted for the greatest percentage of the macrobotanical remains (46%), but they declined sharply to 19% in starch grains. Grasses increased in both the macrobotanical remains (37%) and starch grains (71%), with a similar increase being detected for weeds (18% in the macrobotanical remains). Within the grasses taxa, cereals (including millet, rice, and Job’s-tears) also increased, but had comparatively small percentages (21% in the macrobotanical remains and 38% in starch grains). Although the proportions of USOs and beans increased (10% and 0.3% in starch grains), they remained minor. During the Middle Neolithic (6~5 ka cal BP), fleshy fruits and nuts significantly decreased to 2% in the macrobotanical remains and 0.2% in starch grains. Grasses greatly increased in the macrobotanical remains (83%), but decreased in starch assemblages (36%), while weeds slightly decreased (15%) in the macrobotanical remains. For the grasses group, cereals increased to 46% in the macrobotanical remains and remained at 33% in starch grains. The percentage of USOs noticeably increased to 63.7% in starch grains and predominated the data, whereas that of beans remained low (0.1% in starch grains).

**Fig 7:**
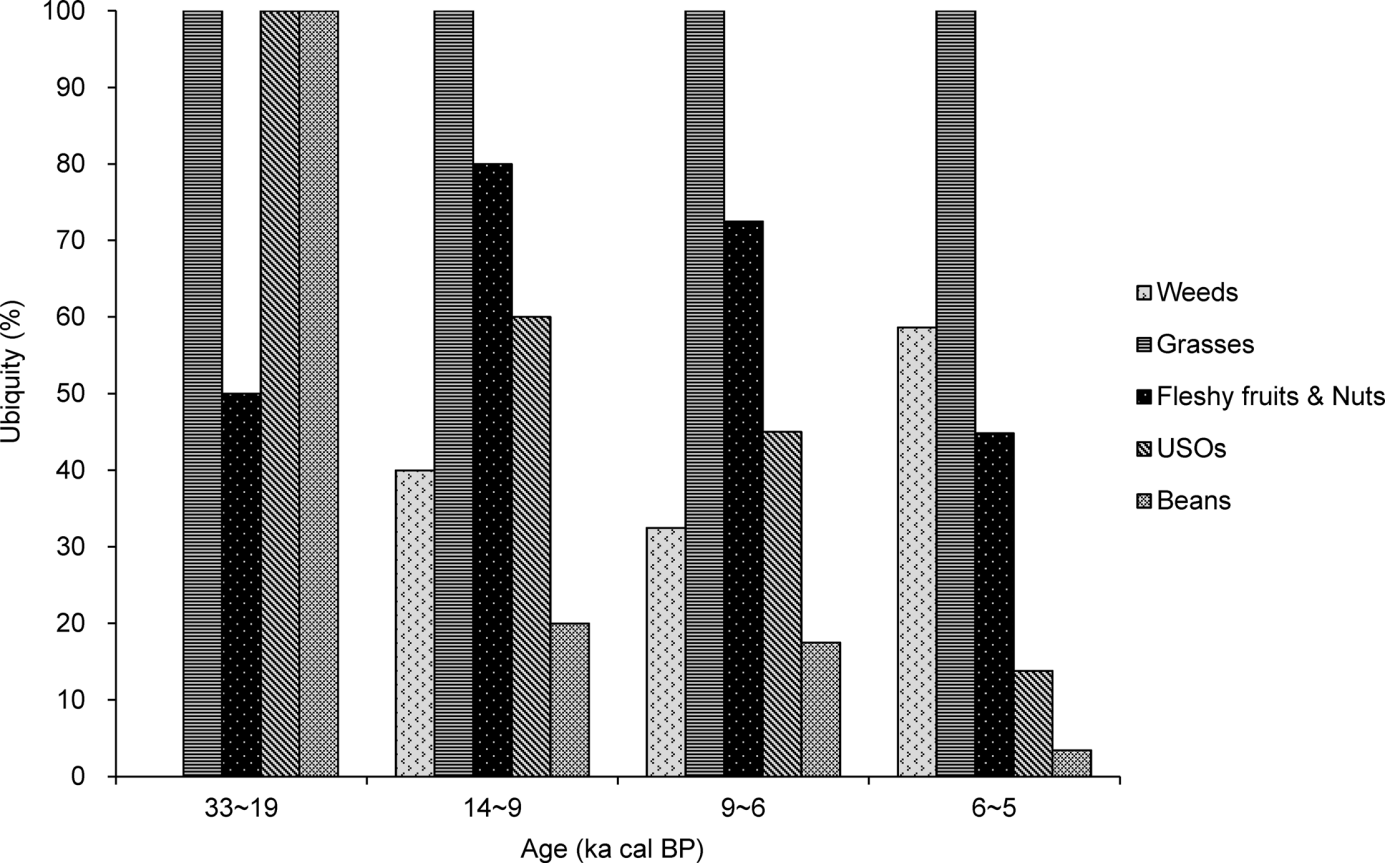
Ubiquity of the 5 plant groups in North China between 33 and 5 ka cal BP.

The percentage ubiquity of grasses in North China was consistently 100% (Fig 7), whereas that of USOs and beans gradually declined from 100% in the Upper Paleolithic period to 13.8% and 3.4% in the middle Neolithic, respectively. The ubiquity of fleshy fruits and nuts was 50% during the Upper Paleolithic period and increased to 80% between 14 and 9 ka cal BP, decreasing to 44.8% in the Middle Neolithic (Fig 7). The ubiquity of weeds was ultimately 58.6% in the Middle Neolithic.

**Fig 8:**
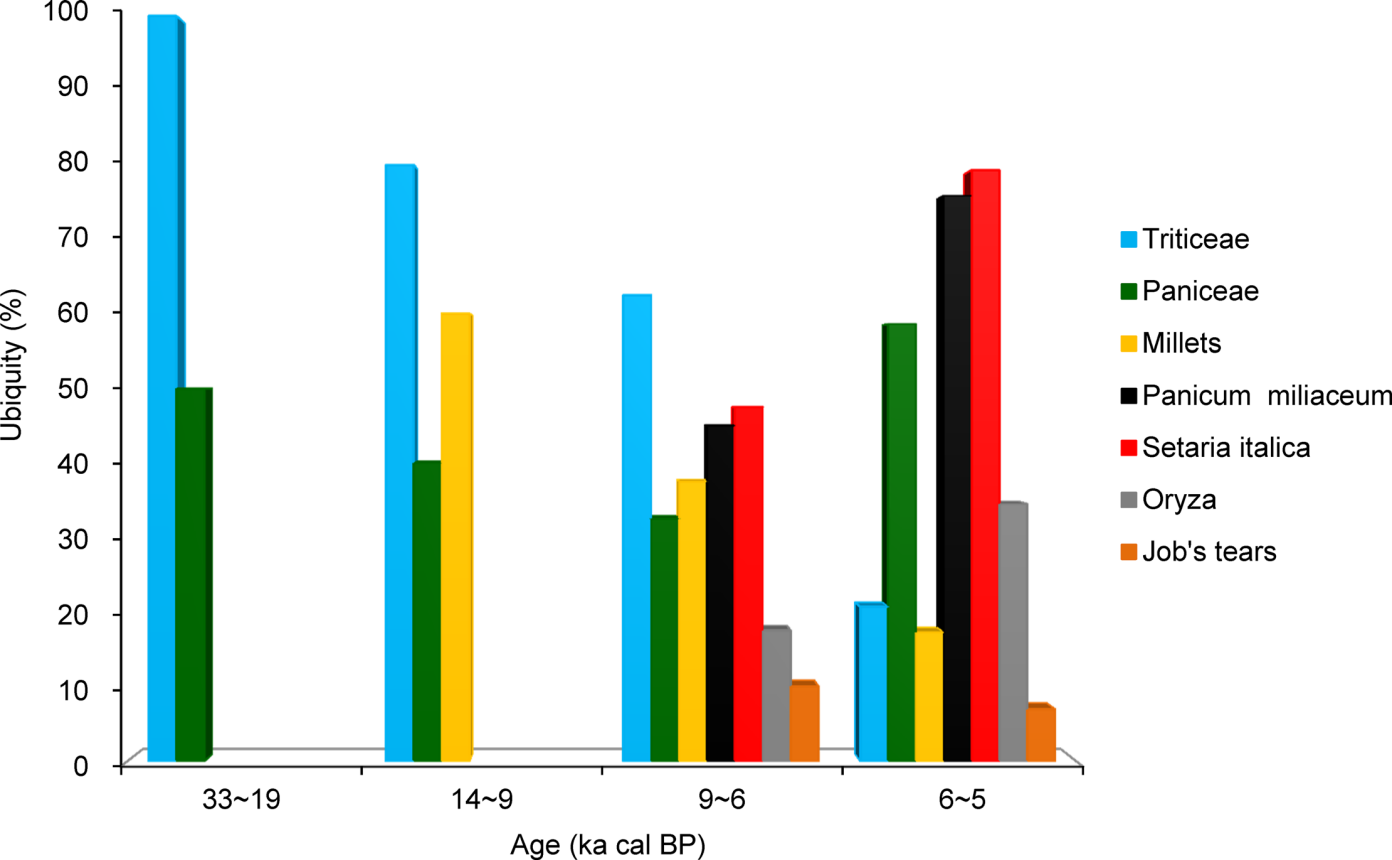
Temporal changes in the ubiquity of several important grasses used in North China in the past.

The temporal changes in the ubiquity of several important grasses used in the past indicate the transition from foraging for wild grasses to cereal-based farming in North China (Fig 8). Wild grasses, such as the tribes Triticeae (100%) and Paniceae (50%), have been exploited since the MIS3 (Marine Isotope Stage 3, 50~26.5 ka cal BP) and LGM periods, dating 33~19 ka cal BP. In the transitional phase, the ubiquity of Triticeae and Paniceae declined to 80% and 40%, respectively. At this time, wild and domesticated millet emerged, with 60% ubiquity. Between 9 and 6 ka cal BP, the ubiquity of domesticated cereals increased, especially foxtail millet (*S*. *italica*) and common millet (*P*. *miliaceum*) with 47.5% and 45.0%, respectively. Yet, Triticeae retained the highest ubiquity, reaching 62.5%. From 6 to 5 ka cal BP, with that of foxtail millet, common millet, and rice markedly increasing to 79.3%, 75.9% and 34.5%, respectively. In comparison, the ubiquity of Triticeae decreased to 20.7%, whereas that of Paniceae reached 58.6%, which may be due to the increased presence of certain Paniceae weeds, such as *Setaria* sp., *Digitaria* sp., along with the popularization of cereals farming.

**Fig 9:**
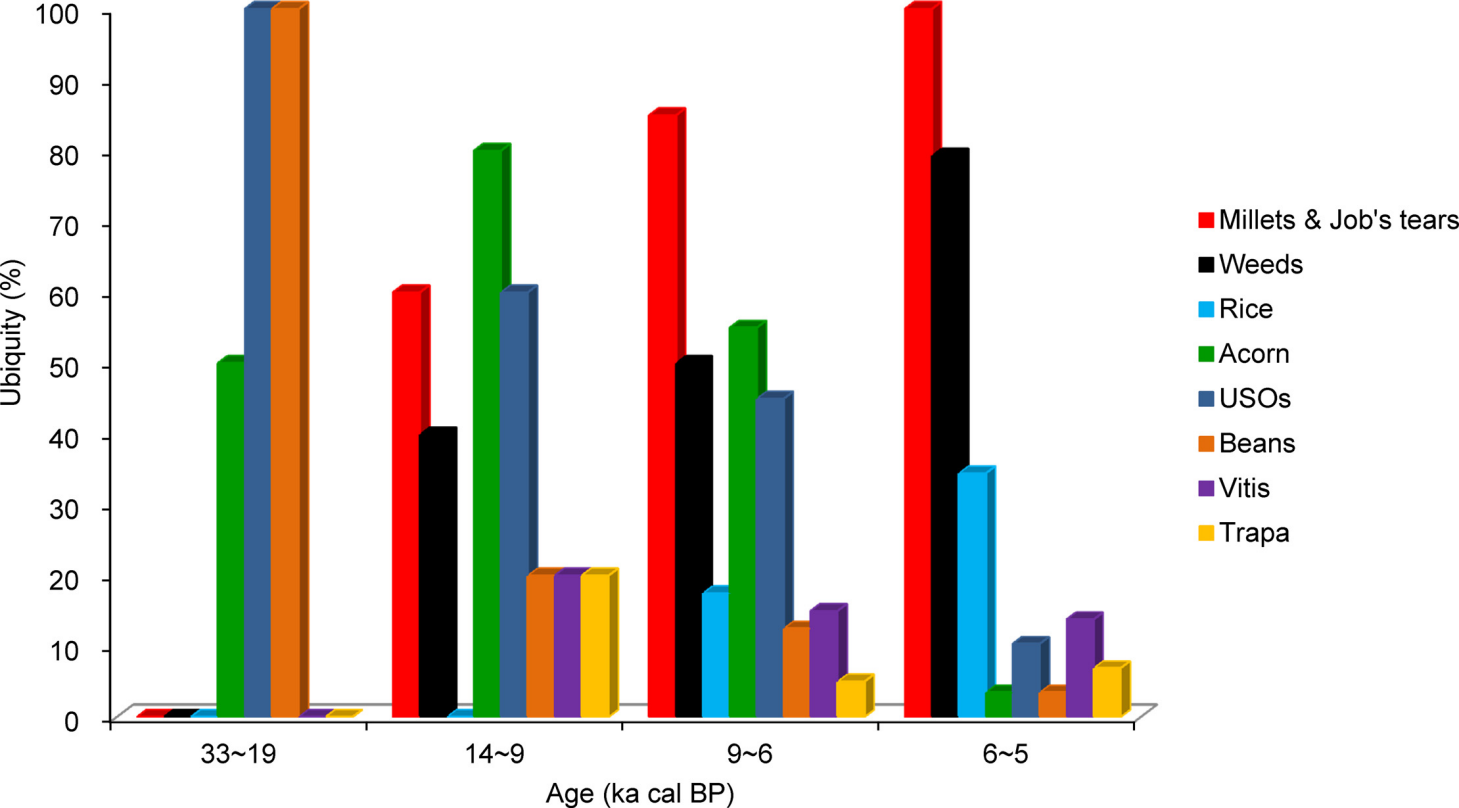
Comparison of the ubiquity for several cereals, weeds, and edible wild plants in North China between 33 and 5 ka cal BP.

Comparison of the ubiquity for several cereals, weeds and edible plants showed that USOs, beans, and acorns were dominant during the Upper Paleolithic (Fig 9). Once cereals and weeds emerged during the transitional phase, their ubiquity gradually increased, whereas that of wild plant groups gradually decreased over time. Finally, between 6 and 5 ka cal BP, cereals (millet, Job's tears, and rice) and weeds reached 34–100% ubiquity, becoming the staples. In contrast, none of the wild plants exceeded 15% ubiquity.

**Fig 10:**
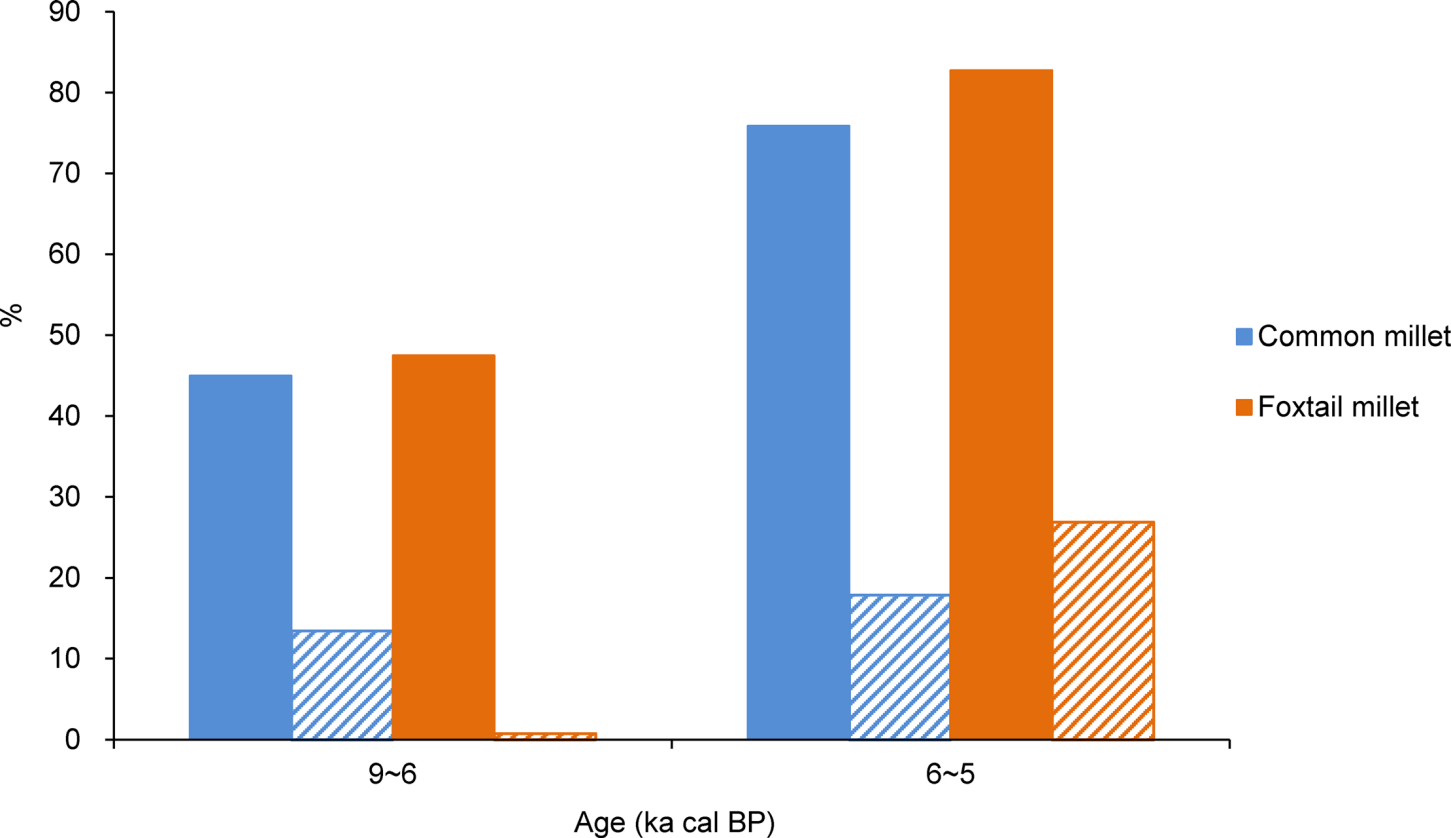
Comparison in the relative percentage (shaded color) and percentage ubiquity (solid color) of foxtail millet and common millet in North China at 9~6 and 6~5 ka cal BP.

Changes in the percentages between the 2 major crops, foxtail millet and common millet, were compared in North China (Fig 10). During the Early Neolithic (9~6 ka cal BP), the percentage of common millet (macrobotanical remains) was 13.4%, while that of foxtail millet was only 0.8%, with both having similar ubiquity (45.0% and 47.5%, respectively). During the Middle Neolithic (6~5 ka cal BP), the percentage and ubiquity of foxtail millet (26.9%; 82.8%) became higher than those of common millet (17.9%; 75.9%), indicating a transition from common millet to foxtail millet as farming became more established after 6 ka cal BP.

## Discussion

Macrobotanical remains recovered by the flotation method are primarily used for understanding past plant use by humans, and represent the basis of several reviews focusing the origins of agriculture in China [26, 30, 42, 43]. Macrobotanical assemblages mainly include the seeds of cereals, weeds, fleshy fruits and nuts; however, these groups represent only a small component of the plants exploited by ancient people. Furthermore, the poor preservation of plant macrofossils means that there is a paucity of published macrobotanical collections dating from the Upper Paleolithic to the transitional phase. Microbotanical remains, such as starch grains and phytoliths, may be preserved in good condition for a long time. Consequently, the analyses of these items are important for the recovery and identification of certain types of ancient plants, including USOs, beans, and early cultivated crops, which rarely survived in macrobotanical remains [58, 59]. Thus, this information complements the list of plants utilized by ancient people. Therefore, in the current study, we used multi-archaeobotanical data to recover more complete plant inventories about past subsistence practices.

Our results indicate that grasses, like the tribes Triticeae and Paniceae, were intentionally collected and processed in both North and South China during the Upper Paleolithic period (ca. 33~19 ka cal BP). Furthermore, these wild grasses, which might have been low in the rankings of foragers [60], appear to have been staple plant foods in the human diet at that time, along with beans and USOs, especially in North China (Fig 6). These observations indicate that the human diet expanded at this time. This phenomenon is similar to the early consumption of grass seeds in western Asia (dated to ca. 23 ka cal BP) [11, 22, 61, 62] and Europe (dated to 30~50 ka cal BP) [12, 13, 63]. However, tribe Triticeae grasses were intensively exploited in the west, leading to a long process of domestication for large-grained cereals, like wheat and barley. In contrast, Paniceae grasses were targeted for foraging in North China, resulting in the cultivation and domestication of some small-seeded crops, like millet, over an extended timeframe. Although Triticeae tribe has always been an important component of past grass use in North China (Fig 8), they retained indigenous wild status throughout use. Grass use during the Upper Paleolithic in China may be related to the dry and cold climate and grass-dominated vegetation during the MIS3 and LGM periods [64–66].

During the transitional phases (ca. 14~9 ka cal BP), fleshy fruits and nuts were the principal plant foods for hunter-gatherer subsistence, which correlated to the gradually warming climate and broadleaf tree expansion during the last deglacial period and the onset of Holocene [66–68]. The pre-domestication cultivation of cereals may have occurred at the same time, along with the collection of wild plants. Even though cereals from South China did not appear in our data (Fig 3), phytolith and/or macrobotanical evidence from Diaotonghuan (~12 ka cal BP) [69, 70], Yuchanyan (~12 ka cal BP) [71–73], Niulandong (~14 ka cal BP) [74], and Shangshan (9~10 ka cal BP) [31, 75, 76] sites indicate that rice was intentionally collected and cultivated as a supplementary resource alongside wild nuts. However, there is insufficient proof for the emergence of domesticated rice at that time. In contrast, wild millet, such as *Setaria* sp., was heavily cultivated, and may have been undergoing domestication in North China. This assumption is indicated by the first presence of morphologically domesticated millet remains at Donghulin [31, 34], Nanzhuangtou [34], Zhuannian [35], and Cishan sites [32] dating to 10~12 ka cal BP. Given this start time for agricultural processes, the cold and dry climate of Younger Dryas (ca. 12.9~11.5 ka cal BP) may have triggered pre-domestication cultivation in China, similar to that suggested for west Asia [24].

The critical transition period from plant gathering to agriculture in China appears to have occurred during the Early Neolithic period (ca. 9~6 ka cal BP). During this period, low-level plant food production based on domesticated cereals occurred in parallel to the continued collection of wild plants, coinciding with the mild climate during the Holocene megathermals [77–80]. The overall subsistence economy was characterized by a mixture of wild resource foraging and farming. In South China, fleshy fruits and nuts, like acorns (including *Quercus*, *Cyclobalanopsis*, and *Lithocarpus*), water chestnuts (*Trapa* sp.), foxnuts (*E*. *ferox*) and persimmon (*Diospyros* sp.), were still used as staples. In comparison, other edible wild plants, such as USOs and beans, might have been used infrequently (Figs 3 and 4), with cereals (primarily rice) representing a small proportion of the subsistence system. However, the consumption of beans, USOs, and rice gradually increased relative to gathered wild plants during this stage (Figs 3 and 4). At this point, the process of rice domestication had begun (Fig 5).

In North China, the gathering of wild plants was also the dominant subsistence strategy. Fleshy fruits and nuts, wild grasses (e.g., Triticeae), and USOs represented another important component of starchy foods in the human diet (Figs 6–8). A large variety of cereals were farmed, including foxtail millet, common millet, rice, and Job's-tears in North China (Fig 8). Thus, food production was more diverse in North China compared to South China. Millet farming was an important component of food production, with common millet being the primary crop cultivated in North China (Fig 10). Millet domestication developed fast, with domesticated species appearing in the records at this time (Fig 8). These species were simultaneously distributed in a range from Shandong to Gansu and from central-south Inner Mongolia to the Central Plain [26]. The increased ubiquity of millet (Fig 9) indicates that the proportion of millet farming in subsistence practices in North China may be relatively higher than that of rice farming in South China.

Over a long timeframe (ca. 30,000 years), the gathering of wild plants declined, while rice and millet agriculture became dominant in subsistence practices between 6 and 5 ka cal BP in both South and North China. This transition is demonstrated by the high percentage of cereal crops and accompanying arable weeds in plant assemblages. Our data also suggest that rice domestication in South China culminated at this stage (Fig 5), while the major crops of dry-land agriculture in North China switched from common millet to foxtail millet (Fig 10), despite the continued farming of common millet at some sites [81, 82]. In addition, differences occurred between the establishment of full rice and millet farming. Within the rice agriculture system of South China, while humans primarily focused on rice cultivation, the collection of nuts (e.g., foxnuts and water chestnuts) remained important in plant subsistence. In contrast, millet agriculture completely replaced gathering practices in North China, with wild edible plants becoming more sporadic and almost disappearing. This establishment of farming as a primary source of food supply in China may have benefited from the favorable climate of the mid-Holocene [83], facilitating the full development of Neolithic cultures [27], the fast development of civilization [84], and rapid population growth [85].

From the above discussions, it is also noticed that the trajectory leading to agriculture can be closely associated with climate change. In general, during the last glacial, cold-dry climatic conditions and low CO_2_ concentrations in atmosphere limited the plant productivity and hence the efficiency of human use of plants [86, 87, 88], ultimately preventing the rise of agriculture in the Upper Paleolithic period. In comparison, climate of the Holocene became warm and wet and comparatively stable [86], making the domestication of crops and the development of agriculture successful in the Neolithic period.

The results of our study make clearly define the 4 stages in the macro-process of plant subsistence from the Upper Paleolithic to Middle Neolithic in China (Table 1). Of note, a gap exists between 19 and 14 ka cal BP; thus, more archaeobotanical studies focusing this time period are needed in the future.

**Table 1:** Stages in the macro-process of plant subsistence from the Upper Paleolithic to the Middle Neolithic in China.

Stage	Time range (ka cal BP)	Cultural period	Characteristics of plant subsistence		Climatic characteristics
			South China	North China	
Ⅰ	33~19	Upper Paleolithic	Intentional grass use	Intentional grass use, along with the use of beans and USOs	Cold and dry
Ⅱ	14~9	Transitional phase	Intensive fleshy fruits and nuts use, accompanied by intentional cultivation of rice	Intensive fleshy fruits and nuts use accompanied by the intentional cultivation of millet; millet was undergoing domestication	Gradual warming
Ⅲ	9~6	Early Neolithic	Fleshy fruits and nuts were used as staples, with an increase in rice consumption; the process of rice domestication had started.	Fleshy fruits and nuts, together with wild grasses and USOs, were dominant; a variety of cereals were cultivated; millet domestication developed very quickly; common millet was the most important crop	Warm and wet
Ⅳ	6~5	Middle Neolithic	Rice farming was dominant; rice domestication culminated; nut collecting remained important in subsistence	Millet farming completely dominated subsistence practices; foxtail millet became the dominant crop; wild plant gathering became more sporadic and almost disappeared	Warm and wet

## Conclusions

Here, we compiled an archaeobotanical database for China from the Upper Paleolithic to the Middle Neolithic period. This information was used to conduct a quantitative analysis of multi-archaeobotanical data to reconstruct the macro-process from hunter-gatherer subsistence to full farming practices. Our results indicate that certain wild plants were intentionally used, in particular grass seeds, since the Upper Paleolithic. Subsequently, the gathering of wild plants always dominated the subsistence system, but gradually declined in importance until the middle Neolithic, dating to 6~5 ka cal BP. At this time, farming based on the domestication of cereals became the major form of subsistence practices in China. Moreover, our analysis showed distinct differences between North and South China in the proportion of certain plant taxa (e.g., Triticeae grass and USOs) in assemblages, the domestication rate of cereals and the type of plant subsistence practiced after the establishment of full farming. Our study indicates that the transition from foraging practices to rice and millet agriculture in China was a slow and long-term process (ca. 30,000 years), which may be analogous to the developmental paths of wheat and barley farming in west Asia. More archaeobotanical research and analysis are required to enhance our understanding about the developmental trajectory of past plant subsistence in China.

## Supporting Information

S1 Dataset The archaeobotanical dataset of China. (XLS)
